# Attitudes about cannabis mediate the relationship between cannabis knowledge and use in active adult athletes

**DOI:** 10.1186/s42238-020-00023-3

**Published:** 2020-05-18

**Authors:** Joanna S. Zeiger, William S. Silvers, Edward M. Fleegler, Robert S. Zeiger

**Affiliations:** 1 Canna Research Group, 3996 Savannah Ct, Boulder, CO 80301 USA; 2 Division of Allergy and Clinical Immunology, University of Colorado Denver School of Medicine, 12700 E. 19th Ave., Room 10C03, Aurora, CO 80045 USA; 3 To-Life in Peace, LLC, 3812 Taft Court, Wheat Ridge, CO 80033 USA; 4 Kaiser Permanente Southern California, 7060 Clairemont Mesa Blvd, San Diego, CA 92111 USA

**Keywords:** Medical cannabis, Cannabis education, Cluster analysis, Athletes, Attitudes, Cannabis

## Abstract

**Background:**

Little is known about how cannabis knowledge and attitudes impact cannabis use behavior.

**Objective:**

To test the knowledge-attitudes-behavior paradigm in active adult athletes.

**Design:**

The Athlete Pain, Exercise, and Cannabis Experience (PEACE) Survey, a cross-sectional survey study, used social media and email blasts to recruit participants and SurveyGizmo to collect data.

**Participants:**

Self-defined active adult athletes (*n* = 1161).

**Main measures:**

Knowledge about cannabis was evaluated with four questions. Attitudes toward cannabis was evaluated with 11 questions. The attitudes questions were used in a TwoStep Cluster analysis in SPSS to assign group membership by attitudes. Chi-square was used to determine if there were differences in cluster membership by demographic factors and if knowledge about cannabis differed by cluster membership. Regression analysis was performed to determine if cannabis attitudes mediated the relationship between cannabis knowledge and cannabis use.

**Key results:**

A three-cluster solution was the best fit to the data. The clusters were named Conservative (*n* = 374, 32.2%), Unsure (*n* = 533, 45.9%), and Liberal (*n* = 254, 21.9). There was a significant difference among the clusters for all 11 attitudes items (all *p* < 0.001). Attitude cluster membership was significantly different by age (*p* < 0.001), primary sport (*p* < 0.05), and knowledge about cannabis (*p* < 0.001). Athletes in the liberal cluster answered the knowledge questions correctly most often. Attitudes mediated the relationship between cannabis knowledge and cannabis use [Never (32.4%), Past (41.6%), Current (26.0%)] with athletes in the liberal cluster showing more knowledge and greater likelihood to be a current cannabis user (*p* < 0.001). Among current cannabis users there were differential patterns of cannabis use depending on their attitudes and knowledge; liberal athletes tended to co-use THC and CBD and used cannabis longer. (*p* < 0.001).

**Conclusions:**

Cannabis education needs to consider attitudes about cannabis, especially among those who might benefit from medical cannabis.

## Background

The inter-relatedness of attitudes and behavior has been evaluated both conceptually and practically (Fazio 1986; Fazio et al. 1983; Bocquier et al. 2005; Bernhardsson et al. 2014). Early theories of the connection between attitudes and behavior were considered “guiding” or “influential” (Fazio et al. 1983) and that this process was one-to-one, ignoring the presence of other potential variables that might impact this relationship (Fazio 1986). It is now widely believed that other factors may change the attitude-behavior relationship with knowledge being one such intervening variable (Fazio 1986; Macaulay et al. 2005; Borden et al. 2008). The relationship between knowledge, attitudes, and behavior is theoretically important, particularly when knowledge and attitudes are impacting deleterious behavior or are needed to boost positive behavior.

The triad of knowledge-attitudes-behavior has not been well studied in terms of cannabis use, but previous studies have taken a harms-avoidance approach examining drug use in adolescents. A study of parenting practices and adolescent drug use tested adolescent drug knowledge, pro-drug attitudes, and adolescent drug use (Macaulay et al. 2005). Pro-drug attitudes predicted both knowledge about drugs and drug use (Macaulay et al. 2005). Another study in adolescents showed that education regarding substance use impacted substance use attitudes which decreased cigarette smoking and cannabis use (Botvin et al. 1990).

The growing popular acceptance of cannabis use among the US population is leading to increasing use, thus warranting the evaluation of the knowledge-attitudes-behavior paradigm (Manzanares et al. 2006; Abuhasira et al. 2018; Green et al. 2003). The four most common reasons for medical cannabis use in a recent study were chronic pain, anxiety, insomnia, and stress with cannabis used as a substitute for prescription drugs (e.g. opiates, anti-depressants, non-opioid pain medicine), alcohol, and illicit substances) (Lucas et al. 2019). Education regarding the therapeutic benefits of cannabis and proper ways to administer cannabis may change attitudes and downstream cannabis use behavior reducing the adverse effects of cannabis (O’Connell et al. 2019).

Despite the growing evidence for the efficacy of cannabis to treat a variety of medical conditions, adverse effects to cannabis are known and educational programs should focus on the potential harms as well as the potential benefits (Zeiger et al. 2019a; Black et al. 2019; Volkow et al. 2014). A recent meta-analysis examining the use of medical cannabis for psychiatric disorders such as ADHD, depression, Tourette Syndrome, and anxiety found that high quality studies are lacking (Black et al. 2019). However, among the studies that were included in the analysis, the authors stated “We found little evidence for the effectiveness of pharmaceutical CBD or medicinal cannabis for the treatment of any of these mental disorders [mentioned above]. Some very-low quality evidence was found for the use of pharmaceutical THC (with or without CBD) in treating anxiety symptoms among individuals with other medical conditions, such as chronic non-cancer pain and multiple sclerosis.” (Black et al. 2019) Furthermore, adverse effects of addiction, impaired motor-coordination, paranoia, psychosis, emergency room visits for vomiting, cognitive impairment, and altered brain development are potential side effects of both short and long term cannabis use (Volkow et al. 2014; Metrik et al. 2011). There has been conflicting evidence around the efficacy of cannabis to alleviate the burden of opioid overdose mortality (Shover et al. 2019).

Cannabis use in athletes has been a contentious issue, as it has been viewed primarily as an area of concern due to anti-doping laws and abuse potential (Ware et al. 2018; Hainline et al. 2017; Brisola-Santos et al. 2016). .Caucasian male athletes who participate in high risk sports (e.g. bobsled, ice hockey, and skeleton) used cannabis more often than other athlete groups and many athletes used cannabis as a means to improve their sports performance (Brisola-Santos et al. 2016). Cannabis has the potential to diminish performance due to reducing alertness and reaction time as well as accelerating muscle fatigue (Gil et al. 2016). However, studies examining both benefits and adverse effects, as well as knowledge and attitudes, regarding cannabis in adult athletes have been overlooked in favor of studies examining risk (Gil et al. 2016; McDuff et al. 2019).

Our study, The Athlete Pain, Exercise, and Cannabis Experience (PEACE) Survey, examined cannabis patterns of use and subjective effects to cannabis in self-defined community-based adult athletes (Zeiger et al. 2019a). Largely, the positive effects of cannabis, including improved sleep, decreased pain, and reduced anxiety, outweighed adverse effects of decreased concentration, increased appetite, and anxiety (Zeiger et al. 2019a). With 49% of athletes indicating they have acute or chronic pain, potential therapies to mitigate the pain are needed; indeed, 69% reported that cannabis reduced their pain (Zeiger et al. 2019a). Age-related differences in patterns of use and subjective effects to cannabis were observed in this population (Zeiger et al. 2019b).

The Athlete PEACE Survey also measured cannabis knowledge and attitudes. The aim of this analysis was to understand the relationship between attitudes, knowledge and cannabis use. We hypothesized that cannabis attitudes mediated the relationship between cannabis knowledge and cannabis use in active adult athletes.

## Methods

This secondary analysis was conducted using data from The Athlete PEACE Survey, a cross-sectional quantitative survey study designed to characterize cannabis use in a convenience sample of self-defined athletes (see paper for survey questions and detailed sample characteristics) (Zeiger et al. 2019a). The survey was administered on SurveyGizmo (https://www.surveygizmo.com) between 6 September 2018 and 7 December 2018 and was approved with waiver of written consent by Solutions IRB (http://www.solutionsirb.com). Participants were assured confidentiality and were informed that “by completing and submitting this survey, you are indicating your consent to participate in the study”. Inclusion criteria included: (1) ages 21 years or older, (2) a self-declared athlete of any sport, and (3) English speaking, with no other inclusions or exclusions. Social media sites such as Facebook, Twitter, and LinkedIn were the primary sources of recruitment. The call to action (Additional file 1) was posted on sports specific Facebook pages as well as on sports specific forums. A web page was set up on a secure website with a link to the study survey. The recruitment material was also posted to endurance sports websites and LinkedIn. There was no paid advertising for the study. Leaflets were left in stores that represented a variety of sports. Email blasts with the call to action were sent out and were shared by other coaches and athletes. The survey module was created to maximize completion of all questions. As such, no data points were missing. The cohort is multi-national (83.5% from the United States) and represents all fifty states. Even though the call-to-action was placed on multiple sport sites, triathletes, cyclists, and runners were the most highly represented athlete groups. Athletes from these sports tend to be early adopters of novel modalities and are very active on message boards, Facebook pages, and forums (Etxebarria et al. 2019; MultiSport Research 2018). All the 1161 subjects in the study analyses completely filled out the questionnaire (Table 1).

**Table 1:** Demographics by cannabis use status in 1161 adult athletes [Data as N (%)]*

Variable	Category	Total (***N*** = 1161)	Current User (***N*** = 302)	Ever, not current User (***N*** = 483)	Never User (***N*** = 376)
Sex^1^	Male	722 (62.2)	182 (60.3)	312 (64.6)	228 (60.6)
	Female	437 (37.6)	120 (39.7)	170 (35.2)	147 (39.1)
Age*	21–39	374 (32.2)	122 (40.4)	139 (28.8)	113 (30.1)
	40 and over	787 (67.8)	180 (59.6)	344 (71.2)	263 (69.9)
Ethnicity	Caucasian	1042 (89.8)	269 (89.1)	439 (90.9)	334 (88.8)
	Other	119 (10.2)	33 (10.9)	44 (9.1)	42 (11.2)
Primary Sport**	Running	299 (25.8)	75 (24.8)	113 (23.4)	111 (29.5)
	Cycling	258 (22.2)	69 (22.8)	111 (23.0)	78 (20.7)
	Triathlon	399 (34.4)	73 (24.2)	184 (38.1)	142 (37.8)
	Other*	205 (17.7)	85 (28.1)	75 (15.5)	45 (12.0)
Days per week exercise**	1–4 days	309 (26.6)	112 (37.1)	116 (24.0)	81 (21.5)
	5–7 days	852 (73.4)	190 (62.9)	367 (76.0)	295 (78.5)
Athlete Status*	Professional	25 (2.2)	11 (3.6)	7 (1.4)	7 (1.9)
	Serious/competitive (amateur)	468 (40.3	100 (33.1)	202 (41.8)	166 (44.1)
	Frequent/fitness athlete	405 (34.9)	100 (33.1)	179 (37.1)	126 (33.5)
	Recreational athlete	243 (20.9)	86 (28.5)	87 (18.0)	70 (18.6)
	Other	20 (1.7)	5 (1.7)	8 (1.7)	7 (1.9)
Pain**	No pain	592 (51.0)	118 (39.1)	261 (54.0)	213 (56.6)
	< 3 months	94 (8.1)	30 (9.9)	34 (7.0)	30 (8.0)
	3 or more months	475 (40.9)	154 (51.0)	188 (38.9)	133 (35.4)
Country**	United States	969 (83.5)	300 (79.8)	396 (82.0)	274 (90.7)
	Other	192 (16.5)	76 (20.2)	87 (18.0)	28 (9.3)
Cannabis legal in state or country**	Yes	583 (50.2)	170 (45.2)	218 (45.1)	195 (64.6)
	No	532 (45.8)	184 (48.9)	247 (51.1)	101 (33.4)
	Unsure	46(4.0)	22 (5.9)	18 (3.7)	6 (2)

*Modified from Zeiger et al. (2019) (Zeiger et al. 2019a); ^1^Not all numbers add to 1161 due to two participants declining to answer the question. Chi-square test for group differences by cannabis use status: *p < 0.01; **p < 0.001

Other sports breakdown: Swimming, 62; Strength training/gym, 29; Trail running, 19; Hiking, 14; Walking, 10; Winter sports (skiing, snowboarding, snow shoeing), 9; Yoga/Pilates, 7; Spartan races, 7; Climbing, 6; Martial arts/MMA, 5; Hockey, 5; Multiple sports, 3; Dance, 3; Soccer, 2; Baseball/, 4; Tennis, 2; Collegiate Soccer/HIIT/Crossfit, 2; Duathlon, 2; Roller derby, 2; Lacrosse, 2; Motor sports / horseback riding, 1; Multiple racquet sports, 1; Pilates, 1; Rebounding, 1; Boxing, 1; Rugby, 1; Sailing, 1; Softball, 1; Rowing, 1; Aquabike, 1; Archery, 1;

There were 113 incomplete surveys in which there was not enough information to impute data or use any of the existing data. Eleven questions regarding cannabis attitudes were used from the questionnaire from the Attitudes on Marijuana Survey conducted by the Hazelden Betty Ford Foundation and Q Market Research (Table 2) (Q Market Research 2015).

**Table 2:** Attitudes toward cannabis in adult athletes by cluster membership. [Data as N (%)]

Attitude question	Response	Conservative (*n* = 374)	Unsure (*n* = 533)	Liberal (*n* = 254)
Do you think marijuana is addictive?±	Yes	228 (61)	125 (23.5)	0 (0)
	No	43 (11.5)	221 (41.5)	254 (100)
	Unsure	103 (27.5)	187 (35.1)	0 (0)
Do you think marijuana is damaging to the brain? ±	Yes	285 (76.2)	131 (24.6)	0 (0)
	No	17 (4.5)	112 (21)	254 (100)
	Unsure	72 (19.3)	290 (54.4)	0 (0)
Do you think consuming edible marijuana is safer than smoking it? ±	Yes	100 (26.7)	324 (60.8)	150 (59.1)
	No	137 (36.6)	62 (11.6)	56 (22.0)
	Unsure	137 (36.6)	147 (27.6)	48 (18.9)
Do you think marijuana is less harmful to your health than alcohol? ±	Yes	38 (10.2)	373 (70)	235 (92.5)
	No	198 (52.9)	12 (2.3)	9 (3.5)
	Unsure	138 (36.9)	148 (27.8)	10 (3.9)
Do you think marijuana is less harmful to your health than tobacco? ±	Yes	77 (20.6)	452 (84.8)	240 (94.5)
	No	179 (47.9)	8 (1.5)	8 (3.1)
	Unsure	118 (31.6)	73 (13.7)	6 (2.4)
Do you think legalizing marijuana makes it seem safer? ±	Yes	176 (47.1)	400 (75)	202 (79.5)
	No	142 (38)	82 (15.4)	39 (15.4)
	Unsure	56 (15.0)	51 (9.6)	13 (5.1)
Do you think marijuana can be beneficial for people with certain medical conditions? ±	Yes	297 (79.4)	524 (98.3)	254 (100)
	No	18 (4.8)	0 (0)	0 (0)
	Unsure	59 (15.8)	9 (1.7)	0 (0)
Do you think legalizing marijuana makes it more socially acceptable? ±	Yes	255 (68.2)	487 (91.4)	246 (96.9)
	No	81 (21.7)	23 (4.3)	5 (2.0)
	Unsure	38 (10.2)	23 (4.3)	3 (1.2)
Do you support the legalization of marijuana for medical purposes? ±	Yes	265 (70.9)	529 (99.2)	254 (100)
	No	46 (12.3)	1 (0.2)	0 (0)
	Unsure	63 (16.8)	3 (0.6)	0 (0)
Do you support the legalization of marijuana for recreational purposes? ±	Yes	64 (17.1)	388 (72.8)	249 (98)
	No	248 (66.3)	45 (8.4)	4 (1.6)
	Unsure	62 (16.6)	100 (18.8)	1 (0.4)
Do you think athletes who use marijuana are doping? ±	Yes	181 (48.4)	47 (8.8)	5 (2)
	No	103 (27.5)	383 (71.9)	231 (90.9)
	Unsure	90 (24.1)	103 (19.3)	18 (7.1)
**Comparisons of Column Proportions**^**b**^				
		TwoStep Cluster Number		
		Conservative	Unsure	Liberal
		(A)	(B)	(C)
Do you think marijuana is addictive?	Yes	B		.^a^
	No		A	.^a^
	Unsure		A	.^a^
Do you think marijuana is damaging to the brain?	Yes	B		.^a^
	No		A	.^a^
	Unsure		A	.^a^
Do you think consuming edible marijuana is safer than smoking it?	Yes		A	A
	No	B C		B
	Unsure	B C	C	
Do you think marijuana is less harmful to your health than alcohol?	Yes		A	A B
	No	B C		
	Unsure	B C	C	
Do you think marijuana is less harmful to your health than tobacco?	Yes		A	A B
	No	B C		
	Unsure	B C	C	
Do you think legalizing marijuana makes it seem safer?	Yes		A	A
	No	B C		
	Unsure	B C		
Do you think marijuana can be beneficial for people with certain medical conditions?	Yes		A	.^a^
	No		.^a^	.^a^
	Unsure	B		.^a^
Do you think legalizing marijuana makes it more socially acceptable?	Yes		A	A B
	No	B C		
	Unsure	B C		
Do you support the legalization of marijuana for medical purposes?	Yes		A	.^a^
	No	B		.^a^
	Unsure	B		.^a^
Do you support the legalization of marijuana for recreational purposes?	Yes		A	A B
	No	B C	C	
	Unsure	C	C	
Do you think athletes who use marijuana are doping?	Yes	B C	C	
	No		A	A B
	Unsure	C	C	

Results are based on two-sided tests. For each significant pair, the key of the category with the smaller column proportion appears in the category with the larger column proportion

Significance level for upper case letters (A, B, C): .05

a. This category is not used in comparisons because its column proportion is equal to zero or one

b. Tests are adjusted for all pairwise comparisons within a row of each innermost sub-table using the Bonferroni correction

Chi-square p-values: ^±^*p* < 0.001

Four questions were asked to measure general knowledge about cannabis (Table 3).

**Table 3:** Knowledge about cannabis in 1161 adult athletes by attitudes cluster. Correct answers are bolded [Data as N (%)]

Knowledge questions	Cannabis type	Conservative (n = 374)	Unsure (n = 533)	Liberal (n = 254)
Which cannabinoid is psychoactive?±	**THC**	203 (54.3)	421 (79)	214 (84.3)
	CBD	3 (0.8)	4 (0.8)	3 (1.2)
	Both	13 (3.5)	5 (0.9)	10 (3.9)
	Neither	5 (1.3)	2 (0.4)	3 (1.2)
	Don’t know	150 (40.1)	101 (18.9)	24 (9.4)
Which cannabinoid has therapeutic benefits that can offer symptom relief to people with pain?±	THC	12 (3.2)	20 (3.8)	6 (2.4)
	CBD	138 (36.9)	262 (49.2)	124 (48.8)
	**Both**	68 (18.2)	165 (31)	99 (39)
	Neither	6 (1.6)	1 (0.2)	0 (0)
	Don’t know	150 (40.1)	85 (15.9)	25 (9.8)
Which cannabinoid is attributed with reducing seizures in patients who suffer from epilepsy?±	THC	32 (8.6)	42 (7.9)	10 (3.9)
	**CBD**	96 (25.7)	227 (42.6)	138 (54.3)
	Both	30 (8.0)	69 (12.9)	49 (19.3)
	Neither	4 (1.1)	2 (0.4)	0 (0)
	Don’t know	212 (56.7)	193 (36.2)	57 (22.4)
The majority of strains available today have been selectively bred for high concentrations of which cannabinoid?±	**THC**	95 (25.4)	236 (44.3)	110 (43.3)
	CBD	44 (11.8)	77 (14.4)	37 (14.6)
	Both	32 (8.6)	54 (10.1)	53 (20.9)
	Neither	1 (0.3)	2 (0.4)	0 (0)
	Don’t know	202 (54.0)	164 (30.8)	54 (21.3)
Mean score (SD)*		1.23 (1.19)	1.97 (1.17)	2.21 (1.10)

Chi-square *p*-values: ^±^*p* < 0.001; *ANOVA *p*-value< 0.001

The SPSS TwoStep cluster analysis procedure was used to create cannabis attitudes groups. Cluster analysis can be used to divide data into smaller groups with similar characteristics when there are no a priori assumptions about differences within the population; it creates homogenous groups within heterogenous data (Ehlert et al. 2017; Punj and Stewart 1983). Cluster analysis was used to identify attitudes phenotypes to determine how these phenotypes relate to knowledge about cannabis and cannabis use. All eleven attitudes questions were used in the cluster analysis.

A systematic analysis of sample sizes for cluster analyses reviewed 243 cluster analyses and found that the median sample size for the cluster analyses was 293 participants (Dolcinar et al. 2014). A simulation study found valid solutions for cluster analysis with samples as small as 20 (Henry et al. 2015). The present sample size of 1161 was adequate for this analysis.

The SPSS TwoStep Cluster is appropriate for large datasets where hierarchical clustering can be cumbersome and difficult to interpret and when the number of clusters is not known a priori. The TwoStep Cluster can determine both the number of clusters and allocate subjects to their respective clusters. The first step is a pre-clustering which uses a sequential clustering approach and then a final clustering using an agglomerative hierarchical clustering method (Bacher et al. 2004). The log-likelihood method with the BIC goodness-of-fit was used whereby a large ratio of distances is considered an optimal number of clusters (Bacher et al. 2004). Once clusters were identified, post-hoc tests were conducted to determine whether there was inter-cluster heterogeneity (i.e. the distribution of subjects per cluster) and intra-cluster homogeneity (chi-square tests to examine cluster separation) (Table 1).

The four knowledge questions had a single correct answer and four incorrect answers. The four question were converted into a knowledge score by summing the four answers, where a correct answer was “1” and an incorrect answer was “0”, with a minimum score of 0 and a maximum score of 4. ANOVA was used to determine whether there were attitudes cluster differences in mean knowledge scores (Table 3).

Cannabis use was measured with two questions: (1) “Have you ever used marijuana?” to which the answers were “yes” or “no” and (2) “In the past two weeks, have you used marijuana (including THC and/or CBD)?” to which the answers were “yes” or “no”. “Never users” answered no to the first question, “Past users” answered yes to the first question and no to the second question and “current users” answered yes to both questions.

**Fig. 1:**
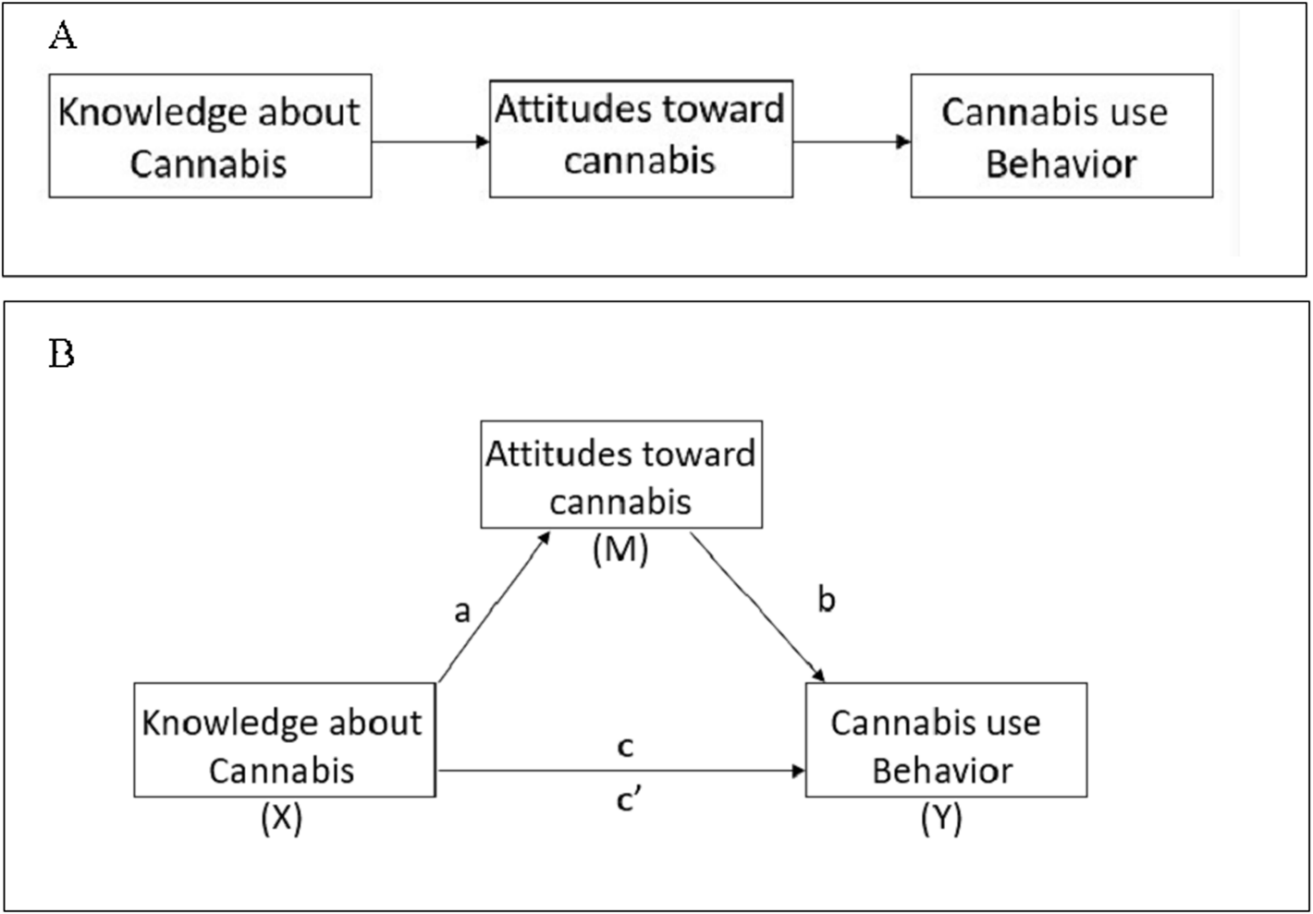
Panel **a** shows Knowledge, Attitudes, Behavior Model. Panel **b** shows the proposed Mediation Model of Knowledge, Attitudes, and Behavior regarding cannabis

Regression analysis was performed to test whether cannabis attitudes mediate the relationship between knowledge and use. Fig. 1, Panel A depicts the Knowledge, Attitude, and Behavior Cognitive Model (Fig. 1, Panel A) (Chatterjee et al. 2009; Valente et al. 1998; Baranowski et al. 2003). This model postulates that first an individual must learn about a behavior, then they develop an attitude toward that behavior which might lead to initiation of a behavior (Valente et al. 1998). The present analysis tests this model using cannabis knowledge, attitudes and use (i.e. behavior) as the measured domains with the potential of attitudes mediating the relationship between knowledge and behavior (Fig. 1, Panel B).

Mediation regression analysis was performed using the PROCESSv3.2 command in SPSS (Hayes 2017). A variable *M* is a mediator when *X* significantly predicts *Y*, *X* significantly predicts M and M significantly predicts *Y* (after controlling for *X*). A mediator variable is in a causal relationship between two variables (MacKinnon et al. 2007). PROCESS examines the direct effect from X (causal variable) to Y (outcome variable) and the indirect effect through M (mediator). The “model 4” designation was chosen. PROCESS calculates the coefficients of 5 paths, from X to M (the “a” path), from M to Y (the “b” path), the total effect (from X to Y without adjusting for the mediator, the “c” path), and the direct effect (from X to Y adjusting for the mediator, the “c’” path) and the indirect effect of X to Y. Direct effects are those effects not impacted by the mediator while indirect effects are the effects which are potentially affected by a mediator (Hayes 2017). If there is mediation, the direct effect should become smaller with the addition of the mediator variable (MacKinnon et al. 2007).

The bootstrapping method of 5000 samples was used to test for the indirect effects; mediation occurs if the 95% confidence intervals estimated by the bootstrapping method do not overlap 0. Results are presented as standardized beta coefficients.

All analyses were conducted in IBM SPSS Statistics for Windows, version 24.0 (2016). Significance was considered at *P* < 0.05, 2-sided.

## Results

### Cluster analysis

A two-cluster solution was automatically designated with a BIC of 17,815.29 (BIC change: − 3172.98). A visual inspection of a three-cluster solution was deemed a better to fit to the data, with a BIC of 16,894.19 (BIC change: − 921.11). A four-class solution was not appropriate based on the BIC change for a three-class solution (− 921.107) vs. a four-class solution (− 556.030). Pairwise comparisons of a four-class solution did not show statistically significant differences between classes 3 and 4 for most of the variables. Table 2 shows the frequencies and chi-square *p*-values for the attitude’s questions by cluster membership; all questions were significantly different between clusters at *p* < 0.001. Furthermore, pairwise comparisons of the proportions show that each cluster is distinct from the other clusters for all of the attitude’s questions. The clusters were named Conservative (*n* = 374, 32.2%), Unsure (*n* = 533, 45.9%), and Liberal (*n* = 254, 21.9%). The clusters were named based on the predominant answers to the questions. The “Conservative” group primarily thought cannabis is addictive (66%), damaging to the brain (76.2%), more harmful than tobacco (47.9%) and alcohol (52.9%), and the majority do not support legalization for recreational purposes (66.3%). This contrasts to the “Liberal” group in which 98% support legalization for recreational purposes, 100% did not think cannabis is addictive or damaging to the brain; and, 92.5 and 94.5% thought alcohol and tobacco, respectively, are more harmful than cannabis. The “Unsure” group fell somewhere in the middle with 35.1% unsure whether cannabis is addictive and 54.4% indicated that cannabis is damaging to the brain and 27.8 and 13.7% were unsure whether cannabis is less harmful to health than alcohol or tobacco, respectively. Almost 19% were unsure whether they support recreational legalization of cannabis.

There were no gender differences between clusters (Table 4), but there were age differences, with younger athletes more often in the Liberal cluster (*p* < 001). Triathletes were the most conservative and athletes in the “Other” group were the most Liberal (*p* < 0.01). Athletes in states or countries in which cannabis use is legal were more often in the Liberal cluster (*p* < 0.05). These variables were added to the mediation models but were not significant and ultimately not included in the final models. Never users of cannabis were more often in the Conservative cluster, Past Users were more often in the Unsure cluster, and Current users were mainly in the Liberal cluster (*p* < 0.001).

**Table 4:** Cannabis use attitudes clusters and demographics in adult athletes [Data as N (%)]

Variables	Category	Cluster		
		Conservative (n = 374)	Unsure (n = 533)	Liberal (n = 254)
Gender	Male	239 (64.1)	326 (61.3)	157 (61.8)
	Female	134 (35.9)	206 (38.7)	97 (38.2)
Age‡	21 to 39	83 (22.2)	186 (34.9)	105 (41.3)
	40 and older	291 (77.8)	347 (65.1)	149 (58.7)
Primary Sport†	Running	103 (27.5)	134 (25.1)	62 (24.4)
	Cycling	92 (24.6)	115 (21.6)	51 (20.1)
	Triathlon	137 (36.6)	185 (34.7)	77 (30.3)
	Other	42 (11.2)	99 (18.6)	64 (25.2)
Level of competitiveness (type of athlete)	Professional	4 (1.1)	13 (2.4)	8 (3.1)
	Serious amateur	169 (45.2)	198 (37.1)	101 (39.8)
	Frequent/fitness	127 (34.0)	203 (38.1)	75 (29.5)
	Recreational	68 (18.2)	110 (20.6)	65 (25.6)
	Other	6 (1.6)	9 (1.7)	5 (2.0)
Hours/week exercise	0–5 h	37 (9.9)	63 (11.8)	22 (8.7)
	6–10 h	172 (46.0)	220 (41.3)	111 (43.7)
	11–15 h	117 (31.3)	169 (31.7)	87 (34.3)
	16–20 h	37 (9.9)	63 (11.8)	23 (9.1)
	more than 20 h	11 (2.9)	18 (3.4)	11 (4.3)
Is marijuana legal in your state or country (if not in the United States)?*	Yes	171 (45.7)	266 (49.9)	146 (57.5)
	No	183 (48.9)	248 (46.5)	101 (39.8)
	Unsure	20 (5.3)	19 (3.6)	7 (2.8)
Cannabis Use‡	Current	13 (3.5)	146 (27.4)	143 (56.3)
	Past Use	141 (37.7)	250 (46.9)	92 (36.2)
	Never	220 (58.8)	137 (25.7)	19 (7.5)

*chi-square *P* < 0.05; ^†^*P* < 0.01; ^‡^*P* < 0.001

### Knowledge measurement

The total mean score for knowledge was 1.78 (SD: 1.22) and the mean knowledge scores for the three clusters were: Conservative (m: 1.24, SD: 1.19), Unsure (m: 1.97, SD: 1.17), Liberal (m: 2.21, SD: 1.10). These differences were statistically significant (F = 65.34, 2 DF, *p* < 0.001). A post-hoc Bonferroni test indicated that all three groups were statistically significantly different from each other at *p* < 0.001 (except for Liberal and Unsure, *p* < 0.05). There was a statistically significant difference in mean knowledge scores by cannabis use status (F = 134.21, 2DF, *p* < 0.001). The means by cannabis use status were: never users (1.21, SD = 1.15), past users (1.72, SD = 1.16) and current users (2.60, SD = 0.95) and a post-hoc Bonferroni test showed that all three groups were significantly different from each other (all *p* < 0.001).

### Mediation

**Fig. 2:**
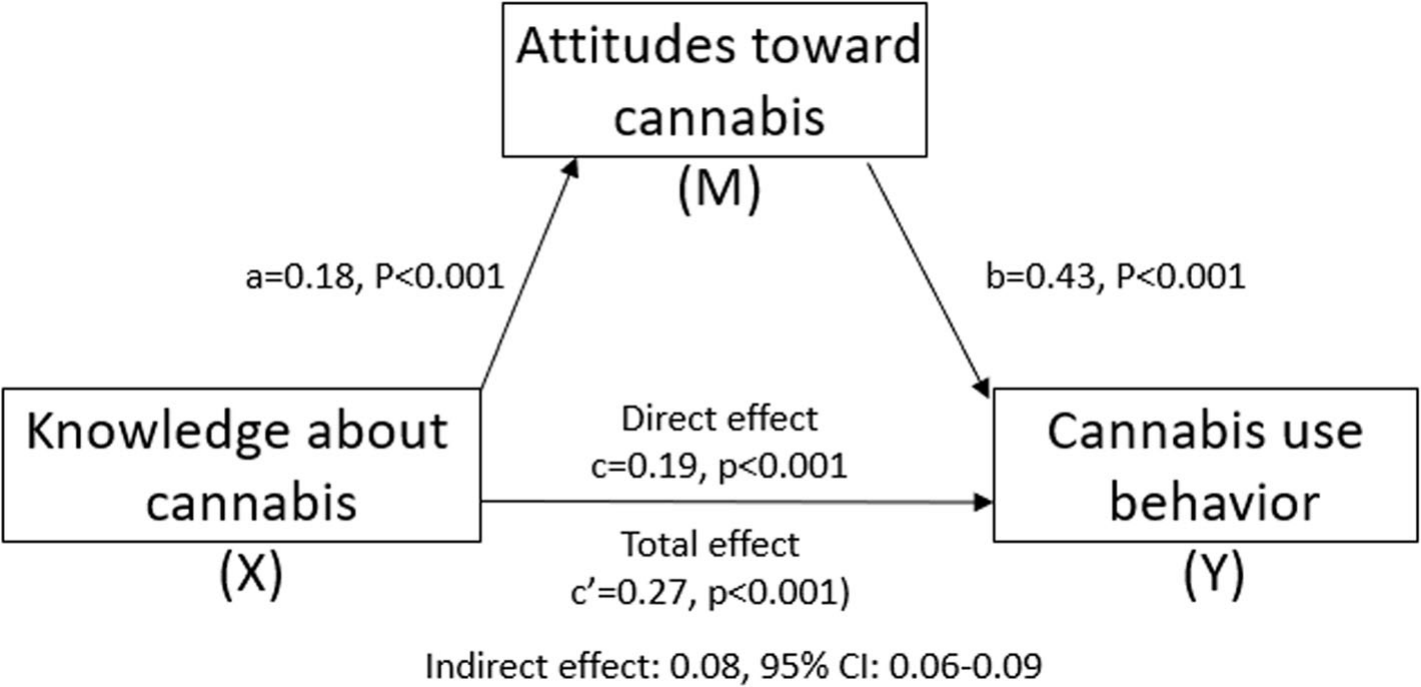
Mediation analysis of attitudes, knowledge, and cannabis use in adult athletes. Effects are standardized coefficients. The X to M path is the effect of knowledge about cannabis on attitudes toward cannabis. Attitudes is coded as 1 = Conservative, 2 = Unsure, 3 = Unsure; Conservative is the comparison group. Knowledge is continuous 0–4. The M to Y path is the effect of attitudes toward cannabis on cannabis use. Cannabis use is coded as: 1 = Never use, 2 = Past Use, 3 = Current Use. The Direct effect is the effect of knowledge on use adjusted for attitudes. The Total effect is the effect of knowledge on use without considering attitudes

The relationship between knowledge about cannabis and cannabis use was significantly mediated by attitudes toward cannabis and explained 34% of the overall variance for cannabis use (R^2^ = 0.34, F(2, 1158) = 293.01, *p* < .001). As Fig. 2 illustrates, the “a” path from knowledge to attitudes was statistically significant (0.18, *p* < 0.001) as was the “b” path from attitudes to cannabis use (0.43, *p* < 0.001). This indicates that athletes with the most knowledge tended to have the most liberal attitudes about cannabis and were more likely to be cannabis users. The direct effect (c = 0.19, *p* < 0.001) and total effect of cannabis knowledge use were statistically significant (c’ = 0.27 (*p* < 0.001). Finally, the indirect effect was 0.08 (95% CI: 0.06–0.09) which was statistically significant, indicating that attitudes toward cannabis mediated the relationship between knowledge about cannabis and cannabis use.

## Discussion

This secondary analysis of the cross-sectional Athlete PEACE Survey study is the first to identify the mediating effect of cannabis attitudes on knowledge and cannabis use. Cannabis knowledge impacted cannabis use directly and indirectly through cannabis attitudes. Athletes were clustered into three attitudes groups: Conservative, Unsure, and Liberal. Liberal and Unsure athletes tended to score better on the knowledge questions and were more likely to be current cannabis users rather than never users or past users compared to the Conservative cluster.

Previous studies regarding the impact of attitudes and knowledge on cannabis use have examined the direct or mediating effects of parental knowledge on their child’s drug use (Macaulay et al. 2005; Sellers et al. 2018; Wu et al. 2015). Parental knowledge indirectly influenced adolescent cannabis use through the number of offers of cannabis (Siegel et al. 2015), when adolescent-reported parental knowledge increased, adolescent alcohol use decreased (Sellers et al. 2018), and maternal knowledge had direct and indirect effects on adolescent substance use (Wang et al. 2009). In adults, attitudes mediated the relationship between information seeking and *intention* to use cannabis, but actual cannabis use was not measured (Martinez and Lewis 2016).

While it is not possible to directly measure causality with cross-sectional studies, research examining Knowledge-Attitude-Behavior relationships have been cross-sectional for practical reasons and to test hypotheses that can guide interventional studies (Valente et al. 1998; Baranowski et al. 2003; Fairchild and McDaniel 2017). Cross-sectional studies are considered appropriate for mediation analysis if confounding is addressed and the temporal relationship of the variables can be established given the measured constructs (Fairchild and McDaniel 2017). We addressed the potential confounders of age, gender, and duration of cannabis use by adding them to the mediation model; none of these variables were significant and were not included in the final model. A review of knowledge, attitudes, and substance use disorder included nine epidemiologic studies which measured all three dimensions contemporaneously and concluded “Lack of knowledge on the risks of substance use has contributed to the increasing cases of substance use disorders. Substance use has been attributed to lack of proper knowledge on the associated risks.” (Njoroge and Kenyatta 2017) With respect to this study, knowledge about cannabis which originates from multiple sources throughout an athlete’s lifetime precedes their attitudes toward cannabis which then impacts their decision making on whether to use cannabis. Certainly, this study is a snapshot, taken at one point in time, whereby all three domains could change by shifting knowledge and/or attitudes (e.g. providing accurate knowledge about cannabis may change an athlete’s attitude and thusly change their behavior).

Medical cannabis has received increased attention due to its potential positive impact on chronic pain, fibromyalgia, inflammatory bowel disease, and other difficult to treat conditions (Bachhuber et al. 2019; Sagy et al. 2019; Ahmed and Katz 2016; Park and Wu 2017). Traditionally, the study of cannabis has been through the lens of reducing use and abuse, with only limited research into benefits from cannabis (Satterlund et al. 2015). Even among the Conservative attitudes cluster in these analyses, 79.4% reported that medical cannabis can be beneficial for people with certain medical conditions and 71% supported medical legalization. Thus, there is still a disconnect between knowledge and attitudes regarding cannabis which could impact behavior, particularly among medical professionals who may be asked by patients to give advice on cannabis use (Berlekamp et al. 2019; Moeller and Woods 2015; Chan et al. 2017).

Given the significant impact of knowledge on cannabis use behavior, the source of knowledge is of utmost importance, particularly given the expanding legalization of cannabis at the state level. Often, the primary source of medical cannabis advice comes from dispensary staff (i.e. budtenders) (Peiper et al. 2017; Wang et al. 2009; Martinez and Lewis 2016). Medical training for budtenders is lacking, with only 20% reporting specific medical or scientific training, yet 94% provided cannabis advice to patients (Haug et al. 2016). This lack of training has seen 69% of surveyed budtenders recommend cannabis products as a treatment for morning sickness in pregnant Colorado women (Dickson et al. 2018), despite the known harmful effects of cannabis use in pregnancy in increasing preterm birth (Corsi et al. 2019). Pharmacists in Minnesota believed there was insufficient training in cannabis pharmacotherapy and were not aware of state-wide regulations, however there was interest in educational programs (Hwang et al. 2016). Pharmacy students poorly identified qualifying medical conditions and adverse effects and they did not feel confident about answering questions about medical marijuana; only 13% indicated formal education on medical marijuana during their training (Moeller and Woods 2015). Colorado medical students had favorable attitudes toward cannabis, however, they did not feel comfortable recommending it, due to lack of evidence and training (Chan et al. 2017).

Underscoring how knowledge and attitudes can potentially affect cannabis use, 12.5% of the Conservative attitudes cluster in this sample indicated they do not use cannabis because they are scared but only 2.7% of the Liberal attitudes cluster chose this as a reason not to use cannabis while the Unsure cluster fell in the middle at 8.3% (this difference was significant, *p* < 0.01). Proper education from informed budtenders and medical professionals could potentially alleviate fear and lack of understanding about the benefits and adverse effects of cannabis. The importance of this undertaking and the need for evidence-based education is clear; unabated or improper cannabis use has increased emergency room visits for cannabis hyperemesis syndrome and psychotic episodes (Monte et al. 2019).

Since this is a convenience sample recruited via multiple sources, it is not possible to know the total number of athletes who saw the call to action and therefore cannot determine the refusal rate. This is a study limitation. The representativeness of this sample to the wider sporting community is unknown. Comparisons to the latest statistics from the governing body of triathlon (USA Triathlon) and cycling (USA Cycling) show that the participant demographics in this sample roughly match the overall populations. The runners in this sample skew male which is different than the sex composition found by Running USA (USA Triathlon 2016; Running 2017; https://legacy.usacycling.org/corp/demographics.php n.d.). Even though the sample demographics roughly reflect those of the greater population of triathletes, runners, and cyclists, the participants are self-selected, therefore the cannabis attitudes clusters and knowledge about cannabis may not be representative of athletes in general. Our data show the following male percentages for the three main sports: 53% running, 81.3% cycling, and 63.45 triathlon. USA Triathlon most recently reported their membership is 65% male (USA Triathlon 2016), while 85.3% of USA Cycling’s member are male (https://legacy.usacycling.org/corp/demographics.php n.d.) and 43% of runners are male (Running 2017). In terms of age of triathletes, 12.8% are 25–29, 35.6% are 30–39, 29.4% are 40–49, and 13.5% are over 50 (USA Triathlon 2016); this compares to our numbers of 14% 21–29, 22.6% 30–39, 28.6% 40–49 and 34.9% over 50. USA Cycling shows that 28.0% of their members are 19–34 and 63.7% are 35 and older (https://legacy.usacycling.org/corp/demographics.php n.d.); 13.5% of our participants who cycled were 21–39. Half of all runners were 25–44 years old (Running 2017) while 62.2% of our runners were over 40.

We recognize that the knowledge and attitudes expressed by this sample may not be representative of other demographic groups. The attitudes observed in this sample were similar to those found in a sample of New York college students (Q Market Research 2015). It is reassuring that the relationship between knowledge, attitudes, and behavior has been observed in other domains (Kuhlemeier et al. 1999; Register-Mihalik et al. 2013; Ozisik et al. 2017). The large sample size allowed for adequate clustering of the data and the mediation regression analysis. In addition, the sample represented a large age-range and varying experience with cannabis use, both of which can impact knowledge and attitudes (Gates et al. 2017; Berlekamp et al. 2019; Moeller and Woods 2015).

It has been noted that cross-sectional data may not be appropriate for mediation analysis, particularly if a process is changing over time (O’Laughlin et al. 2018). The Knowledge-Attitude-Behavior model postulates the temporal ordering, as was succinctly stated by Baranowski et al.: “As knowledge accumulates in a health behavior domain, changes in attitude are initiated. Over some period of time, changes in attitude accumulate, resulting in behavioral change.” (Baranowski et al. 2003) Furthermore, the present analysis provides important insights: (1) cannabis epidemiology is a nascent field, thus obtaining information to direct future epidemiologic studies is an essential first step; (2) longitudinal studies are not necessarily the only way to examine the Knowledge-Attitude-Behavior relationship and often are not feasible to conduct (Buckner et al. 2019; Ball et al. 2018; Stubbs et al. 2018; Buckner et al. 2018); and (3) future interventional study can test this model.

In conclusion, these mediation analyses indicated that cannabis attitudes appeared to mediate the effect of knowledge on use. As more states legalize medical and recreational cannabis, proper consumer, budtender, and medical professional education will become paramount to the safety and efficacy of cannabis use.

## Supplementary information

**Additional file 1.** Call to Action for The Athlete PEACE Survey

## Data Availability

The datasets used and/or analyzed during the current study are available from the corresponding author on reasonable request.

## Abbreviations

THC: Tetrahydrocannabinol
CBD: Cannabidiol
The Athlete PEACE Survey: The Athlete Pain, Exercise, and Cannabis Experience Survey
KAB model: Knowledge-Attitudes-Behavior
