# Supplementary material for: Attitudes about cannabis mediate the relationship between cannabis knowledge and use in active adult athletes
Source: J Cannabis Res. 2020 May 18;2:18. doi: 10.1186/s42238-020-00023-3 (PMC7819330; doi:10.1186/s42238-020-00023-3)
Supplement: Supplementary file 1 — Additional file 1. Call to Action for The Athlete PEACE Survey [file 42238_2020_23_MOESM1_ESM.docx]

Additional File 1: Call to Action for The Athlete PEACE Survey

The Athlete PEACE Survey

(Pain, Exercise, And Cannabis Experience)

Calling all athletes! We are looking for you to be part of an important research study about cannabis consumption, knowledge, and attitudes in athletes. It doesn’t matter how fast you are, how long you have been participating in sports, what sport you do, or how many hours per week you exercise. If you are getting out there and exercising, you are an athlete.

• Are you over the age of 21?

• Are you an English-speaking athlete?

If you answered YES to these questions, you might be eligible to participate in a research survey.

Cannabis consumption, including THC and CBD, has increased in athletes to help pain, recovery, and sleep. We want to understand these trends better. Participants will be asked to complete an anonymous 8-minute questionnaire. We hope there will be direct benefits to participants when our results become available and you will be contributing to the growing field of cannabis research.

https://www.surveygizmo.com/s3/4539059/The-Athlete-PEACE-Survey

This survey is being conducted by Joanna Zeiger, M.S., Ph.D., Olympian and World Champion. Please contact Joanna at joannazeiger@comcast.net for more information or questions
